# Modeling of Mouse Experiments Suggests that Optimal Anti-Hormonal Treatment for Breast Cancer is Diet-Dependent

**DOI:** 10.1007/s11538-023-01253-1

**Published:** 2024-03-18

**Authors:** Tuğba Akman, Lisa M. Arendt, Jürgen Geisler, Vessela N. Kristensen, Arnoldo Frigessi, Alvaro Köhn-Luque

**Affiliations:** 1 Oslo Centre for Biostatistics and Epidemiology, Faculty of Medicine, University of Oslo, 0317 Oslo, Norway; 2 Department of Computer Engineering, University of Turkish Aeronautical Association, 06790 Etimesgut, Ankara Turkey; 3 Department of Comparative Biosciences, University of Wisconsin-Madison, Madison, WI USA; 4 Department of Oncology, Akershus University Hospital, Lørenskog, Norway; 5 Institute of Clinical Medicine, Faculty of Medicine, University of Oslo, Campus AHUS, Oslo, Norway; 6 Department of Medical Genetics, Institute of Clinical Medicine, Oslo University Hospital and University of Oslo, Oslo, Norway; 7 Oslo Centre for Biostatistics and Epidemiology, Oslo University Hospital, Oslo, Norway

**Keywords:** Optimal control, Differential equations, Estrogen receptor positive breast cancer, Aromatase inhibitors, Drug resistance, High-fat diet

## Abstract

Estrogen receptor positive breast cancer is frequently treated with anti-hormonal treatment such as aromatase inhibitors (AI). Interestingly, a high body mass index has been shown to have a negative impact on AI efficacy, most likely due to disturbances in steroid metabolism and adipokine production. Here, we propose a mathematical model based on a system of ordinary differential equations to investigate the effect of high-fat diet on tumor growth. We inform the model with data from mouse experiments, where the animals are fed with high-fat or control (normal) diet. By incorporating AI treatment with drug resistance into the model and by solving optimal control problems we found differential responses for control and high-fat diet. To the best of our knowledge, this is the first attempt to model optimal anti-hormonal treatment for breast cancer in the presence of drug resistance. Our results underline the importance of considering high-fat diet and obesity as factors influencing clinical outcomes during anti-hormonal therapies in breast cancer patients.

**Supplementary Information:**

The online version contains supplementary material available at 10.1007/s11538-023-01253-1.

## Introduction

Lifestyle factors such as age at menarche and menopause, body mass index, child birth and breast feeding, as well as genetic disposition, among others, are well-established breast cancer risk factors (Wu et al. 2016; Neuhouser et al. 2015). However, much less is known about the role lifestyle factors play on breast cancer treatment response. Anti-hormonal treatment for estrogen receptor (ER) positive breast cancer constitutes a puzzling case in obese patients that requires more quantitative investigation. Approximately 75% of all breast tumors express ER, and most women with these tumors will receive anti-hormonal therapy (Clark et al. 1984). ER in breast cancer cells is activated by estrogen and it promotes cell proliferation and tumor growth (Johnston and Dowsett 2003). Anti-hormonal treatment with Aromatase Inhibitors (AI) decreases estrogen levels while anti-estrogen’s block directly the action of steroids at the estrogen receptor (Pearson et al. 1982). Interestingly, high Body Mass Index (BMI) and adiposity have a negative impact on AI efficacy (Folkerd et al. 2012; Ioannides et al. 2014; Jiralerspong and Goodwin 2016; Bahrami et al. 2021; Wang et al. 2015; Gelsomino et al. 2020; Goodwin and Pritchard 2010; Lønning et al. 2014; Sendur et al. 2012). While the puzzle of the optimal anti-hormonal therapy in postmenopausal obese women is still unfinished, good monitoring of the suppression of estrogen levels with valid methods may guide treatment decisions during treatment with aromatase inhibitors (Bordeleau et al. 2010; Ligibel et al. 2012).

An additional layer of complexity arises from the fact that ER-positive breast cancer cells may be resistant to anti-hormonal treatments. Resistance can arise due to multiple mechanisms that are not completely understood (Daldorff et al. 2017; Ma et al. 2015). Tumor cells can adapt to AI therapy after exposure for certain time (adaptive resistance), for instance due to the upregulation of ER expression or activation of alternative pathways conferring the cells survival and proliferative capacity. Instead, de novo or pre-existing resistance refers to the presence of estrogen independent cells before therapy. For instance, cells carrying specific mutations of the ER that confer constitutive ligand-idependent activity (Jeselsohn et al. 2015), which might lead to clonal selection under anti-hormonal treatment. The current paradigm consist of administering high AI doses to both obese and non obese patients, but this may not be the best strategy to avoid or delay drug resistance.

The aforementioned issues are difficult to quantify in preclinical and clinical settings and can benefit from more formal approaches. Here, we propose a new mathematical model, based on a system of ordinary differential equations (ODEs), to model the concentration of estrogen in the cancer tissue, which takes into account the local interplay between the tumor and fat tissues. We inform the model with data from mouse experiments that investigate the effect of obesity in breast cancer using two groups of mice, fed with control diet (CD) or with high-fat diet (HFD). Then, we incorporate AI therapy into the calibrated models, including de novo and adaptive resistance. To determine optimal therapeutic interventions in the CD and HFD cases, we formulate an optimal control problem (OCP) with the goal of minimizing the total tumor volume and the total amount of treatment that is used. We also compare the obtained optimal schedules with constant and alternating treatments.

Mathematical modeling of breast cancer dynamics under treatment have gained interest for long time (Norton and Simon 1977; Enderling et al. 2006, 2007; Frieboes et al. 2009; Roe-Dale et al. 2011; Yankeelov et al. 2013; Lai et al. 2018; Jarrett et al. 2019; Lai et al. 2019, 2022). However, modeling of AI treatment in ER-positive breast cancer has received less attention so far. For example, an ODE model was proposed to understand pathway dynamics of ER-positive MCF-7 breast cancer cells under combination of Cdk4/6 inhibition and anti-hormonal therapies, including AI treatment (He et al. 2020). Similarly, Chen et al. proposed a mathematical model based on a system of ODEs to understand resistance to AI treatment driven by a shift from estrogen to growth factor receptors (Chen et al. 2013). In another study that uses stochastic differential equations and statistical physics techniques, the transitions under AI treatment between three different estrogen sensitive phenotypes were considered (Chen et al. 2014). To explain the dual effect of estrogen inducing both growth and regression of hormone-dependent breast cancer (referred as estrogen paradox), Ouifki and Oke proposed an ODE model and determined conditions to eliminate cancer recurrence for long-term treatment based on stability analysis (Ouifki and Oke 2022). Cancer treatment scheduling optimization by means of OCPs has received considerable attention (Schättler and Ledzewicz 2015; Jarrett et al. 2020; Akman Yıldız et al. 2018a, b). For instance, OCPs were proposed to optimise treatment schedules of chemotherapies (De Pillis and Radunskaya 2001; Panetta and Fister 2003; Ledzewicz and Schättler 2022), angiogenic inhibitors (Ledzewicz and Schättler 2007), cytotoxic and antiangiogenic therapies (Colli et al. 2021), immunotherapy via a dendritic cell vaccine (Castiglione and Piccoli 2007) and combination therapies (Ledzewicz and Schättler 2012; Sharp et al. 2020). In addition, resistance to chemotherapy (Costa et al. 1992; Carrere 2017) or combination of chemotherapy with ketogenic diet (Oke et al. 2018) were also studied using OCPs. Another recent study investigated the optimal combination of doxorubicin and HER2 targeting drug trastuzumab, for a murine model of human HER2 positive breast cancer (Lima et al. 2022). To the best of our knowledge, the present work is the first modeling study to account for anti-hormonal treatment using AIs in the presence of drug resistance in an optimal control framework.

The paper is organized as follows: In the following Sect. 2, we formulate the dynamical model, prove some basic properties and we proceed with model calibration. Section 3 is dedicated to the model extension for AI treatment and resistance. In Sect. 4, we formulate the OCP and derive the optimality system. Then, we proceed with results in Sect. 5 to compare various relevant scenarios for anti-hormonal treatment. We conclude by discussing the main conclusions, limitations and future directions.

## Mathematical Model Development and Calibration

In this section, we develop a basic ODE model for the interaction of ER-positive breast tumor cells, estrogen and fat for the postmenopausal situation, and demonstrate some useful mathematical properties of its solution. Our model can describe the contribution of fat intake differences to estrogen and tumor growth over time. We have been inspired by the mice experiment conducted by Hillers et al. (2018) comparing tumor growth in CD and HFD mice, and we use the data obtained in that study to bring our model closer to reality. We proceed by describing the experiments and available data that inspired our model. We then present model equations and the assumptions they are based on. Then, we discuss mathematical properties of the model. Lastly, we explain the details of the model calibration.

### Experimental Data

Hillers et al. investigated the influence of obesity on breast tumor size and stromal cells within the mammary adipose tissue (Hillers et al. 2018). We use data from that study that utilized breast cancer cell line EO771 derived from a spontaneous mammary adenocarcinoma from a C57Bl/6 mouse. EO771 cells are considered to be a model of luminal B breast cancer subtype and are known to respond to anti-estrogens (Le Naour et al. 2020). Specifically, mice were fed with CD (10% kcal from fat, Test Diet 58Y1) or HFD diet (60% kcal from fat, Test Diet 58Y2). A total of $1 \times 10^6$ EO771 tumor cells were mixed with $2.5 \times 10^5$ adipocytes taken from CD or HFD mice. After pelleting this mixture of cancer and fat cells, it was injected bilaterally into the inguinal mammary glands of 8-week-old female mice fed with CD. In total, we have the data of eleven mice, five where the fat cells come from mice fed with CD and six where fat cells come from mice fed with HFD. For each tumor independently, tumor volumes were measured at days 10, 13 and 15, as depicted in the study (Hillers et al. 2018, Fig. 2B). In addition, the number of adipocytes at day 15 was quantified, see Hillers et al. (2018, Fig. S2F), and we use it to estimate the amount of fat in the tumor tissue. Details are provided in the Supplementary Material. Mice were euthanized when tumor reached the humane endpoint of 15 mm in diameter. In summary, we obtained the initial conditions and six independent measures of tumor volume at three time points (days 10, 13 and 15) for each condition (CD and HFD), and two data points for fat volume at day 15, one for each condition (CD and HFD).

### Model Development

In order to quantify the effect that the fat-induced production of estrogen has on tumor growth, we model the temporal dynamics of tumor volume $T:=T(t)$ (mm$^3$), estrogen concentration $E:=E(t)$ (pg/g) and fat volume $F:=F(t)$ (mm$^3$) in the tumor tissue at time *t* (days). The model is based on the following six assumptions:

1. Tumor volume follows logistic growth (Benzekry et al. 2014).
2. Tumor growth rate depends on the estrogen level (Le Naour et al. 2020).
3. Fatty tissue is the major source of estrogen in the tumor (Simpson 2003). Circulating estrogen concentrations are proportional to adipose mass in postmenopausal women (Marchand et al. 2018), so we assume that estrogen is produced by fat at a constant rate.
4. Estrogen is washed out from the tumor tissue at a constant rate (Deshpande et al. 1967).
5. Tumor cells use fat as an energy resource (Hoy et al. 2017; Wang et al. 2017).
6. While mice are fed CD for 15 days, there is no growth source for fat volume. Therefore, diet-based difference in fat volume (CD vs HFD) are accounted for simply due to the amount of fat volume at day 0.
7. In the experiments that we model, most implanted adipocytes survive. Cancer cells are known to produce growth factors and cytokines that support the survival of adipocytes. Therefore, we did not include fat decrease due to adipocyte death.

**Fig. 1:**
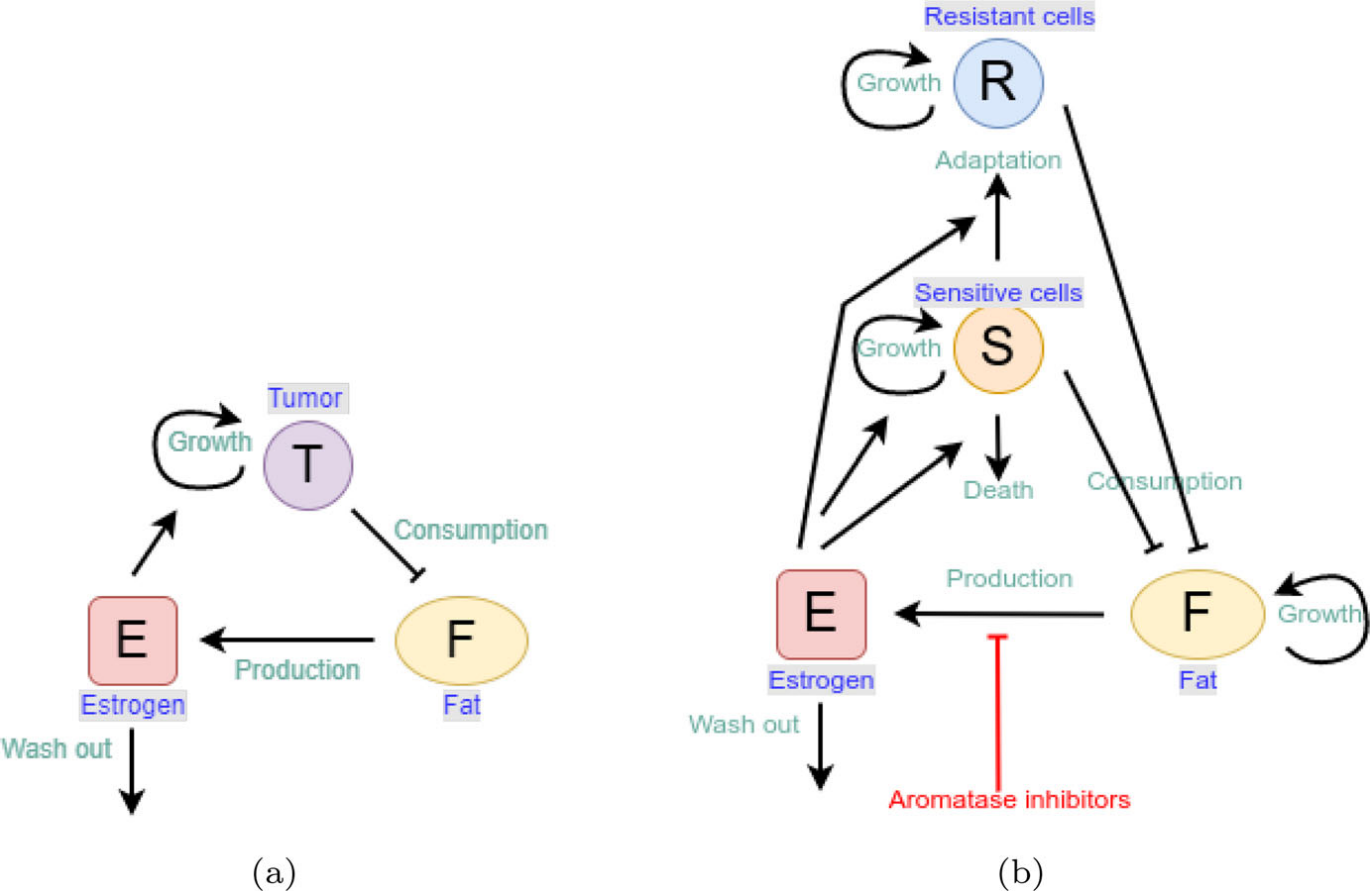
**a** Modelled interactions between the volume of tumor cells, *T*, fat volume, *F*, and estrogen concentration, *E*, in Eq. (2.1). Tumor volume grows as a consequence of cancer cell proliferation, which is triggered by estrogen. Estrogen is produced by fat and it is washed out. Tumor cells consume fat as energy resource. **b** Modelled interactions between the volume of resistant cells, *R*, volume of sensitive cells, *S*, fat volume, *F*, and estrogen concentration, *E*, in Eq. (3.1). The volume of both sensitive and resistant cells grows as a consequence of cell proliferation. While the growth of sensitive cells is triggered by estrogen, that of resistance cells is estrogen independent. Sensitive cells can die under the influence of estrogen or can adapt to low estrogen levels and become resistant. Both sensitive and resistant cells consume fat. Fat volume can change size as a consequence of diet. Estrogen is produced by fat but this production is inhibited by AIs. Estrogen is also naturally washed out. In both diagrams, the lines ending with an arrow represent positive feedback whereas the lines ending with a bar denotes negative feedback

A flow diagram depicting the interactions between model variables *T*, *E* and *F* is presented in Fig. 1a.

Consequently, we propose the following system of ODEs:

2.1a $$\begin{aligned} \underbrace{\dfrac{dT}{dt}}_{change~ in~ tumor~ volume}&= \underbrace{\overbrace{\frac{k_1 E}{a_1 + E}}^{proliferation~rate~triggered~by~estrogen} T \Big (1- m_1 T \Big )}_{logistic~growth~term} , \end{aligned}$$

2.1b $$\begin{aligned} \underbrace{\dfrac{dE}{dt}}_{change~ in~ estrogen~ concentration}&= \underbrace{rF}_{estrogen~production} - \underbrace{\mu E}_{wash~out}, \end{aligned}$$

2.1c $$\begin{aligned} \underbrace{\dfrac{dF}{dt}}_{change~ in~ fat~ volume}&= \underbrace{- \alpha TF}_{energy~consumption}, \nonumber \\ T(0)&=T_0, \, E(0) = E_{0}, \quad F(0)= F_{0}. \end{aligned}$$

The parameters $k_1, a_1, m_1, r, \mu ,\alpha$ and initial conditions $T_0, E_{0}$ and $F_{0}$ are all non-negative real numbers. Equation (2.1a) represents the tumor logistic growth, where the growth rate is assumed to follow Michaelis-Menten kinetics, $g(E)=\frac{k_1 E}{a_1 + E}$. Parameter $k_1$ is the maximum growth rate for high estrogen levels and $a_1$ is the estrogen concentration at which the growth rate is half-maximum. Parameter $m_1$ is the inverse carrying capacity of the tumor. Equation (2.1b) models the change in estrogen concentration when it is produced by fat at a rate *r* and washed out from the tumor tissue at a rate $\mu$. The last equation (2.1c) accounts for fat consumption by tumor cells at a rate $\alpha$. The values of these parameters are not known and will be estimated from data.

### Model Properties

Next we prove that the solution to model (2.1) exists, it is unique, non-negative and bounded. These properties will be used later.

#### Proposition 1

Equation (2.1) with non-negative initial conditions has a unique solution that is non-negative and bounded from above for all $t\ge 0$.

#### Proof

As the right-hand side of the model (2.1) and their partial derivatives are continuous on ${\mathbb {R}} \times {\mathbb {R}}^3$, it follows from the Cauchy-Lipschitz theorem that the existence and uniqueness of the solution are guaranteed (Schatzman 2002, Ch.15).

To prove that the solution to Eq. (2.1) is non-negative for all $t\ge 0$, we use the method of separation of variables. Firstly, Eq. (2.1c) leads to

2.2 $$\begin{aligned} F(t) = F(0) \exp \left( - \int _{0}^{t} \alpha T(s) \, ds \right) \ge 0. \end{aligned}$$

Since *F* and *r* are non-negative, we can rewrite Eq. (2.1b) as

2.3 $$\begin{aligned} \dfrac{dE}{dt} \ge - \mu E. \end{aligned}$$

Thus, $E \ge 0$. Finally, Eq. (2.1a) leads to

2.4 $$\begin{aligned} T(t) = T(0) \exp \left( \int _{0}^{t} \frac{k_1 E(s)}{a_1 + E(s)} \left( 1 - m_1 T(s) \right) \, ds \right) \ge 0. \end{aligned}$$

Therefore, $T \ge 0$, $E \ge 0$ and $F \ge 0$ for all $t \ge 0$.

To prove that the solution to Eq. (2.1) is bounded from above, we observe from Eq. (2.1a) that

2.5 $$\begin{aligned} \frac{dT}{dt} \le k_1 T \Big (1- m_1 T \Big ). \end{aligned}$$

Then,

2.6 $$\begin{aligned} \lim _{t \rightarrow \infty } \sup m_1 T(t) \le \frac{1}{m_1}. \end{aligned}$$

Since $\frac{dF}{dt} \le 0$, *F* stays constant at $F_0$ or decreases. Then, $F(t) \le F_0$. Finally, Eq. (2.1b) leads to

2.7 $$\begin{aligned} \frac{dE}{dt} \le r F_0 - \mu E, \end{aligned}$$

and

2.8 $$\begin{aligned} \lim _{t \rightarrow \infty } \sup E(t) \le \frac{r F_0}{\mu }. \end{aligned}$$

Thus, the solution is bounded from above. $\square$

### Model Calibration

In this section, we make use of the experimental data described in Sect. 2.1 to inform our basic model. Direct measures of model parameters are not available in this experimental setup, and we do not have enough data to do formal statistical inference for all parameters. The main argument we used to fix some parameters was identifiability of the remaining free parameters. To that goal, we decided to fix three of them to reasonable values and made extra assumptions to fix the initial estrogen concentration and fat volume. We then use the available data to calibrate the rest of the parameters for which we lack any information and show that the problem is practically identifiable by using profile likelihood (Kreutz et al. 2012).

The initial amount of fat in the tumor tissue was measured only at day 15. For simplicity, we assume that the level of fat under CD stays constant and it has not changed since the beginning of the experiment (see Hillers et al. 2018, Fig. S2F). We acknowledge this is a limitation and an initial fat measurement would have made our results more solid. As estrogen is mainly produced by fat, we also assume that estrogen concentration is proportional to fat volume at baseline. Indeed, estrogen concentration in mice under different, but comparable, conditions was measured between 150 and 1500 pg/g (Yue et al. 1999, Fig. 2). For estrogen concentration in our model to lie within those measures, we assume that the ratio of estrogen concentration to fat volume is around 3.4.

We then find reasonable values for parameters $m_1$ and $\mu$. We obtain the half-life of estrogen in breast tumor tissue from Deshpande et al. (1967), $t_{1/2}$=2.8 h. Therefore, $\mu$, that represents the washout rate of estrogen from tumor tissue, can be computed as $\mu =\ln (2)/t_{1/2}=0.25$ h$^{-1}=5.94$ day$^{-1}$. In the experiments, mice were euthanized after the tumor reached 15 mm in diameter, corresponding to a volume of approximately 1767 mm$^3$ assuming a spherical tumor. We simply set $m_1=1/2000$ mm$^{-3}$ equally for CD and HFD as a larger value than the highest tumor volume in the data set. Fixing $m_1$ and $\mu$ still did not solve the non-identifiability problem, but we discovered that fixing *r* in addition solved this issue. Parameter *r* is fixed as 20 pg/g mm$^{-3}$ day$^{-1}$ based on the assumption that estrogen is at the steady-state in the beginning of the experiment which leads to $E = \frac{rF}{\mu }$. Estrogen concentration roughly satisfies $150 \le E= \frac{rF}{\mu } \le 1500$ (Yue et al. 1999, Fig. 2). By multiplying both sides of this inequality by $\mu =5.94$, we reach $891 \le r F \le 8910$. We divide both sides by $F_{CD}(0)=50$ and $F_{HFD}(0)=360$ separately, that results in two inequalities $17.82 \le r \le 178.2$ and $2.475 \le r \le 24.75$. The intersection of these inequalities gives a range for the parameter *r* which is $17.82 \le r \le 24.75$. Therefore, we simply choose $r=20$.

We perform model calibration and profile likelihood calculations in Data2Dynamics (Raue et al. 2015, 2013). We fixed the lower and upper bounds for the parameters as $10^{-7}$ and $10^4$ in the optimization problem, respectively. Based on the method of profile likelihood (Kreutz et al. 2012), our model is practically identifiable (See. 7). We list the obtained parameter values in Table 1. We perform sensitivity analysis for all the parameters in Supplementary Material..

**Table 1:** Values of the parameters in the Eq. (2.1)

Parameter	Description	Units	Value
$m_1$	Inverse carrying capacity of tumor	mm$^{-3}$	1/2000 (assumed)
$\mu$	Estrogen washout rate	day$^{-1}$	5.94, Deshpande et al. (1967)
$k_1$	Tumor growth rate	day$^{-1}$	0.55 (calibrated)
$a_1$	Half maximum estrogen threshold	pg/g	43 (calibrated)
*r*	Estrogen production rate	pg/g mm$^{-3}$ day$^{-1}$	20 (assumed)
$\alpha$	Fat consumption rate	day$^{-1}$ mm$^{-3}$	1.7e$-$06 (calibrated)
$T_{0}$	Initial tumor volume	mm$^{3}$	1, Hillers et al. (2018)
$E_{0}$	Initial estrogen concentration	pg/g	170, CD (estimated)
			1200, HFD (estimated)
$F_{0}$	Initial fat volume	mm$^{3}$	50, CD (assumed)
			360, HFD (assumed)

**Fig. 2:**
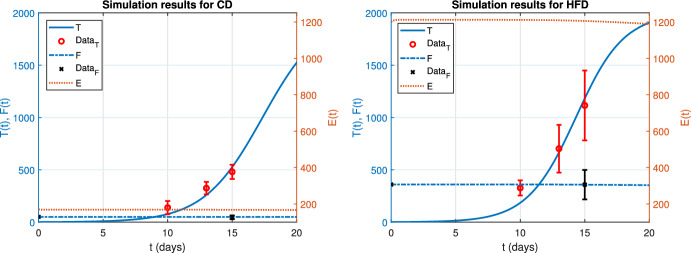
Simulation results of the Eq. (2.1) for CD (*left*) and HFD (*right*) with the data points. Left axis corresponds to tumor size *T*(*t*) and fat volume *F*(*t*), right axis denotes estrogen concentration *E*(*t*) over time *t* (color figure online)

Figure 2 shows the simulation results for CD (left panel) and HFD (right panel) obtained with the parameters in Table 1. The *y* left-axes correspond to tumor or fat volume, whereas the *y* right-axes denote the estrogen level. Data points with error bars for tumor and fat volume are marked with red circles and black crosses, respectively. We observe that simulation results for tumor and fat volume agree well with the data, whereas estrogen level stays within a biologically meaningful interval. We observe that the initial estrogen level and fat volume are higher for HFD than CD (see, Table 1). Temporal evolution of estrogen level and fat volume is similar for both diet types, since fat is assumed as the source of estrogen production. Indeed, tumor associated with HFD increases faster than for the other, due to more estrogen release from HFD fat volume.

Original model (2.1) expresses the changing dynamics of tumor volume, estrogen level and amount of fat in case of no treatment and simulation results agree well with the available data. The next step is to extend this model to account for AI treatment by considering sensitive and resistant tumor subpopulations. In this way we will be able to study drug resistance to endocrine therapy for ER-positive breast cancer.

## Model Extension for Resistance to Aromatase Inhibitor Treatment

Aromatase inhibitors, despite of being an effective treatment choice for ER-positive breast cancer, may suffer from drug resistance (Chumsri et al. 2011; Ma et al. 2015). To investigate different treatment schedules, including constant, alternating and optimal anti-hormonal treatment, we consider tumor heterogeneity in terms of sensitive and resistant subpopulations under the following assumptions:

1. Breast cancer cells are either sensitive or resistant to estrogen deprivation with AIs. In reality, there could be more than two tumor subpopulations, since development of resistance is considered as a progressive mechanism and cells may shift form one stage to another over time (Normanno et al. 2005). For simplicity, we assume that there are only two tumor subpopulations, called sensitive and resistant.
2. Both sensitive and resistant populations follow logistic growth (Benzekry et al. 2014).
3. Growth of sensitive cells is triggered by estrogen (Doisneau-Sixou et al. 2003).
4. Sensitive cells die under low estrogen concentrations (Doisneau-Sixou et al. 2003).
5. Sensitive cells adapt to low estrogen levels and become resistant cells (Chen et al. 2013, 2014).
6. Resistant cells do not die under low estrogen concentrations (Chen et al. 2013, 2014).
7. Fat volume follows logistic growth (Ku-Carrillo et al. 2016).
8. Both sensitive and resistant cells consume fat as energy resource (Ku-Carrillo et al. 2016).

Consequently, Eq. (2.1) is extended with the sensitive cell population $S:=S(t)$ (mm$^3$) and the resistant cell population $R:=R(t)$ (mm$^3$) in the tumor tissue at time *t* (days):

3.1a $$\begin{aligned} \underbrace{\dfrac{dS}{dt}}_{change~in~sensitive~cell~population}&= \underbrace{ \frac{k_1 E}{a_1 + E} S\Big (1- m_1 ( S + \eta R ) \Big )}_{logistic \, growth \, term} - \underbrace{\frac{c a_2^l}{a_2^l + E^l} S}_{death \, term} - \underbrace{\frac{c a_3^l}{a_3^l + E^l} S}_{adaptation \, term} , \end{aligned}$$

3.1b $$\begin{aligned} \underbrace{\dfrac{dR}{dt}}_{change~in~resistant~cell~population}&= \underbrace{k_3 R \Big (1- m_1 ( S + \eta R ) \Big ) }_{logistic \, growth \, term } + \underbrace{\frac{c a_3^l}{a_3^l + E^l} S}_{adaptation \, term} , \end{aligned}$$

3.1c $$\begin{aligned} \underbrace{\dfrac{dE}{dt}}_{change~in~estrogen~concentration}&= \underbrace{ pr F}_{estrogen \, production} - \underbrace{\mu E}_{wash \, out}, \end{aligned}$$

3.1d $$\begin{aligned} \underbrace{\dfrac{dF}{dt}}_{change~in~fat~volume}&= \underbrace{k_2 F (1-m_2 F)}_{logistic \, growth \, term} - \underbrace{ \alpha (S+R)F}_{energy \, consumption} ,\nonumber \\ S(0)&=S_0, \, R(0)=R_0, \, E(0) = E_{0}, \, F(0)= F_{0}, \end{aligned}$$

with the non-negative initial conditions $S_0$, $R_0$, $E_0$ and $F_0$. Equation (3.1a) expresses the logistic growth of sensitive cells over time together with death and adaptation terms. Parameter $m_1$ is the inverse of maximum tumor size and $\eta$ is the competition parameter scaling inhibition of sensitive cells’ growth by resistant cells. Sensitive cells die if estrogen level is smaller than $a_2$ while they adapt to estrogen level below $a_3$ and become resistant. Parameter *c* is the maximum death rate and *l* denotes Hill’s coefficient. Equation (3.1b) models evolution of resistant cells with the growth rate $k_3$. Equation (3.1c) stands for dynamics of estrogen level where the parameter *p*, $0<p\le 1$, reduces the effect *r* to $p \cdot r$ due to aromatase inhibitors. Equation (3.1d) models the change in fat volume with logistic growth so that effect of fat growth to anti-hormonal treatment could be investigated. Parameters $k_2$ and $m_2$ are the growth rate of fat and inverse carrying capacity of fat, respectively. The carrying capacity could model how the body is prone to accumulate fat depending on the life style or other metabolic conditions. In addition, both sensitive and resistant cells consume fat as energy resource at the rate $\alpha$. A diagram depicting the interactions between the extended model variables *S*, *R*, *E* and *F* is presented in Fig. 1b.

We assume that the parameters that we calibrated in the basic model are not affected by the treatment and we use them in the extended model. As we do not have data under treatment, we explore the effect that new parameters have by testing different values.

### Model Properties

#### Proposition 2

Equation (3.1) with non-negative initial conditions has a unique solution that is non-negative and bounded from above for all $t\ge 0$.

#### Proof

Existence and uniqueness of the solution is standard and analogous to Proposition 1. Thus, we prove here that the solution to Eq. (3.1) is non-negative and bounded from above for $t \ge 0$. Similar to Theorem 1, we can prove that $E, F \ge 0$ for $t\ge 0$ by the variation of constants formula. For Eq. (3.1a), we have

3.2 $$\begin{aligned} S(t)&= S(0) \exp \Bigg \{ \int _{0}^t \bigg (\frac{k_1 E(s)}{a_1 + E(s)} \bigg (1- m_1 ( S(s)+ \eta R(s) ) \bigg ) \nonumber \\&\quad - \frac{ca_2^l}{a_2^l + E^l(s)} - \frac{ca_3^l}{a_3^l + E^l(s)}\bigg ) \Bigg \} \ge 0. \end{aligned}$$

Since $S \ge 0$ for $t\ge 0$, Eq. (3.1b) can be written as

3.3 $$\begin{aligned} \dfrac{dR}{dt} \ge k_3 R \Big (1- m_1 ( S(s)+ \eta R(s) ) \Big ). \end{aligned}$$

Then, we get

3.4 $$\begin{aligned} R(t) \ge R(0) \exp \Big \{ \int _0^t k_3 \Big (1- m_1 ( S(s)+ \eta R(s) ) \Big ) \; ds \Big \} \ge 0. \end{aligned}$$

We can prove that *F* and *E* are bounded from above similar to Theorem 1. On the other hand, using Eq. (3.1a)-(3.1b), we obtain the sum

3.5 $$\begin{aligned} \frac{dS}{dt} + \eta \frac{dR}{dt}&\le \underbrace{ \frac{k_1 E}{a_1 + E}}_{\le k_1} S\Big (1- m_1 ( S(s)+ \eta R(s) ) \Big ) + \eta k_3 R \Big (1- m_1 ( S(s)+ \eta R(s) ) \Big ) \nonumber \\&\le (k_1 S + \eta k_3 R) \Big (1- m_1 ( S(s)+ \eta R(s) ) \Big ) \nonumber \\&\le \max \{ k_1, k_3 \} (S + \eta R) \Big (1- m_1 ( S(s)+ \eta R(s) ) \Big ). \end{aligned}$$

Thus,

3.6 $$\begin{aligned} \lim _{t \rightarrow \infty } \sup (S(t) + \eta R(t)) \le 1/ m_1. \end{aligned}$$

Since *S* and *R* are non-negative, it means that *S* and *R* are bounded above. Then, we complete the proof. $\square$

### Treatment Modelling

We will investigate differences between constant, intermittent and optimal anti-hormonal treatment. Constant treatment is implemented through the parameter $0 \le p \le 1$ in Eq. 3.1. The value $p=1$ corresponds to no estrogen deprivation treatment and smaller values of *p* models AI treatment with inhibition of estrogen production.

Alternating treatment refers to a pre-scheduled treatment scenario with $u_{I}:=u_{I}(t)$ and it is implemented by modifying Eq. (3.1c) to

3.7a $$\begin{aligned} \dfrac{dE}{dt}&= (1-u_{I}) r F - \mu E, \end{aligned}$$

where

3.7b $$\begin{aligned} u_{I} = {\left\{ \begin{array}{ll} u_b, \hbox { where } 0 \le u_b < 1, \quad \hbox {if treatment is applied}, \\ 0, \quad \hbox { else}, \end{array}\right. } \end{aligned}$$

In the next section, an OCP is constructed to investigate the optimal value of *p* as a time-dependent function, and results obtained with the optimal endocrine therapy are compared with the constant and alternating treatment.

## Optimal Control Problem for Anti-Hormonal Treatment

We aim to investigate optimal AI treatment schedules that minimize the total number of cancer cells together with the pharmaceutical intervention over a prespecified time interval $[t_{tr},t_{f}]$. We do not include an equation representing the drug as often done for optimal chemotherapy scheduling in the literature (see, for example, de Pillis et al. 2008; Sharma and Samanta 2016). Instead, we model the effect of AI treatment through a continuous control function $u:=u(t)$. AIs act by lowering the estrogen production, so we replace the parameter *p* in Eq. (3.1c) by the function $1-u$.

We formulate the OCP as follows: minimize the cost functional

4.1 $$\begin{aligned} {\mathcal {J}}(u) = \int _{t_{tr}}^{t_{f}} ( \omega _S S + \omega _R R + \frac{\omega _u}{2} u^2 ) \; dt, \end{aligned}$$

subject to

4.2a $$\begin{aligned} \dfrac{dS}{dt}&= \frac{k_1 E}{a_1 + E} S\Big (1- m_1 ( S+ \eta R) \Big ) - \frac{ca_2^l}{a_2^l + E^l} S - \frac{ca_3^l}{a_3^l + E^l} S , \end{aligned}$$

4.2b $$\begin{aligned} \dfrac{dR}{dt}&= k_3 R \Big (1- m_1 ( S+ \eta R ) \Big ) + \frac{ca_3^l}{a_3^l + E^l} S, \end{aligned}$$

4.2c $$\begin{aligned} \dfrac{dE}{dt}&= (1-u) r F - \mu E, \end{aligned}$$

4.2d $$\begin{aligned} \dfrac{dF}{dt}&= k_2 F (1-m_2 F) - \alpha (S+R)F , \nonumber \\ S(0)&=S_0, \, R(0)=R_0, \, E(0) = E_{0}, \, F(0)= F_{0}, \end{aligned}$$

where,

4.3 $$\begin{aligned} {\mathcal {U}} = \{ u \, \mid u \hbox { is\,measurable,} \, u_a \le u \le u_b, \hbox { for all } t \in [t_{tr}, t_{f}], t_{tr} \ge 0, t_{f} >0 \}. \end{aligned}$$

Our aim is to find an optimal control $u^{*}$ such that ${\mathcal {J}}(u^{*}) = \min _{u \in {\mathcal {U}}} {\mathcal {J}}(u)$.

We note that constructions of linear or quadratic cost functional in the control function *u* results in not only biologically but also mathematically different interpretations. While quadratic OCPs have a single extremum and result in continuous controls, linear OCPs result in bang-bang controls and mathematical analysis becomes more complicated due to singular or bang-bang controls that result in non-differentiable solutions curves. We refer readers to the following studies for a detailed comparison (Ledzewicz et al. 2004; Sharp et al. 2019; Ledzewicz and Schättler 2020). In addition, the parameters $\omega _S$, $\omega _R$ and $\omega _u$ in Eq. (4.1) can be set to balance the size of the different terms.

In the present case, inclusion of the term $u^2$ in the Eq. (4.1) is justified by the treatment side effects. Side effects of AI include from hot flushes to cardiovascular events, vaginal bleeding and bone loss (Osborne and Tripathy 2005; Cuzick 2005; Hadji 2010). Our quadratic choice reflects the fact that the increase in side effects is negligible for small amounts of therapy and that side effects increase as function of u, rather than increasing at a constant rate as in the linear control.

### Theorem 1

There exists an optimal control $u^{*}$ with a corresponding solution $(S^{*}, R^{*}, E^{*}, F^{*})$ to the model (4.2) with non-negative initial conditions that minimizes (4.1) over ${\mathcal {U}}$.

### Proof

The proof is based on several steps according to the study of Fleming and Rishel (1975, Corollary 4.1). Firstly, we observe that the coefficients in Eq. (4.2) and its solution are bounded on a finite time interval, so the admissible control set ${\mathcal {U}}$ and the corresponding solution with initial conditions are non-empty (Lukes 1982, Thm 9.2.1.). Secondly, the admissible control set ${\mathcal {U}}$ is closed and convex. In addition, the right-hand side of the system (4.2), namely $\textbf{f}(t,\textbf{X},u)$ with $\textbf{X}=(S,R,E,F)^{T}$, is continuous, since the system has positive parameters and the non-negative solution by Proposition 2. Indeed, it is bounded above by a linear combination of the bounded control and the state as

4.4 $$\begin{aligned} \mid \textbf{f}(t,\textbf{X},u) \mid&= \Big |\begin{pmatrix} \frac{k_1 E}{a_1 + E} S\Big (1- m_1 ( S+ \eta R ) \Big ) - \frac{ca_2^l}{a_2^l + E^l} S - \frac{ca_3^l}{a_3^l + E^l} S \\ k_3 R \Big (1- m_1 ( S+ \eta R ) \Big ) + \frac{ca_3^l}{a_3^l + E^l} S \\ (1-u) r F - \mu E \\ k_2 F (1-m_2 F) - \alpha (S+R)F \end{pmatrix} \Big |\nonumber \\&\le |\begin{pmatrix} k_1 &{} 0 &{} 0 &{}0 \\ 0 &{} k_3 &{} 0 &{} 0 \\ 0 &{} 0 &{} -\mu &{} r\\ 0 &{} 0 &{} 0 &{} k_2 \end{pmatrix} \begin{pmatrix} S\\ R\\ E \\ F \end{pmatrix} |+ |\begin{pmatrix} 0\\ 0\\ \frac{r}{m_2} u \\ 0 \end{pmatrix} |\nonumber \\&\le C( \mid \textbf{X} \mid + \mid u \mid ), \end{aligned}$$

due to bounded solution (by Proposition 2) and positive parameters in the model for some positive constant *C*. On the third line, we use the relation

4.5 $$\begin{aligned} (1-u)rF - \mu E \le (1+u)rF - \mu E \le rF - \mu E + \frac{r}{m_2}u. \end{aligned}$$

The integrand of the objective functional is convex on ${\mathcal {U}}$ due to the quadratic term. Indeed, it is bounded as

4.6 $$\begin{aligned} \omega _S S + \omega _R R + \frac{\omega _u}{2} u^2 \ge \frac{\omega _u}{2} u^2 \ge - {\hat{C}} + \frac{\omega _u}{2} u^2, \end{aligned}$$

with some positive constant ${\hat{C}}$. Thus, we can conclude that an optimal control $u^{*}$ exists. $\square$

### Theorem 2

Given an optimal control $u^{*}$ and solution to the system (4.2) for (4.1), there exist adjoint variables $\lambda _i:= \lambda _i(t)$ for $1 \le i \le 4$ such that

4.7a $$\begin{aligned} \frac{d \lambda _1}{dt}&= - w_S - \lambda _1 \left\{ \frac{k_1 E}{a_1 + E} \Big (1 - m_1 ( 2S + \eta R ) \Big ) - \frac{ca_2^l}{a_2^l + E^l} - \frac{ca_3^l}{a_3^l + E^l} \right\} \nonumber \\&+ \lambda _2 \left\{ m_1 k_3 R + \frac{ca_3^l}{a_3^l + E^l} \right\} + \lambda _4 \alpha F, \end{aligned}$$

4.7b $$\begin{aligned} \frac{d \lambda _2}{dt}&= - w_R + \lambda _1 \left\{ \frac{k_1 m_1 \eta E S }{(a_1 + E)} \right\} - \lambda _2 \left\{ k_3 \Big (1- m_1 (S + 2 \eta R)\Big ) \right\} + \lambda _4 \alpha F, \end{aligned}$$

4.7c $$\begin{aligned} \frac{d \lambda _3}{dt}&= - \lambda _1 \left\{ \! S \Big ( 1- m_1 ( S(s)+ \eta R(s) )\Big ) \frac{ k_1 a_1 }{(a_1 + E)^2} + \Big ( \frac{ca_2^l}{(a_2^l + E^l)^2} + \frac{ca_3^l}{(a_3^l + E^l)^2} \Big ) l E^{l-1}S\! \right\} \nonumber \\&+ \lambda _2 \left\{ \frac{ca_3^l}{(a_3^l + E^l)^2} l E^{l-1} S \right\} + \lambda _3 \mu , \end{aligned}$$

4.7d $$\begin{aligned} \frac{d \lambda _4}{dt}&= - \lambda _3 (1-u^{*})r - \lambda _4 (k_2 - 2k_2 m_2 F - \alpha (S+R)), \end{aligned}$$

with

4.7e $$\begin{aligned} \lambda _i(t_f) = 0, \quad 1 \le i \le 4. \end{aligned}$$

Furthermore, $u^{*}$ can be represented by

4.8 $$\begin{aligned} u^{*} = \min \Big (u_b, \max \Big ( u_a, \frac{r F \lambda _3}{\omega _u} \Big ) \Big ). \end{aligned}$$

### Proof

Following references (de Pillis et al. 2008; Burden et al. 2004), the Lagrangian is constructed as

4.9 $$\begin{aligned} {\mathcal {L}} = {\mathcal {H}} + \xi _1(t) (u - u_a) - \xi _2(t) (u_b-u), \end{aligned}$$

where the Hamiltonian ${\mathcal {H}}$ is defined as

4.10 $$\begin{aligned}&{\mathcal {H}}(S, R, E, F, \lambda _1, \lambda _2, \lambda _3, \lambda _4, u) \nonumber \\&\quad := ( w_S S + w_R R + \frac{\omega _u}{2} u^2 ) \nonumber \\&\qquad + \lambda _1 \Big ( \frac{k_1 E}{a_1 + E} S\Big (1- m_1 ( S+ \eta R ) \Big ) - \frac{ca_2^l}{a_2^l + E^l} S - \frac{ca_3^l}{a_3^l + E^l} S \Big ) \nonumber \\&\qquad + \lambda _2 \Big ( k_3 R \Big (1- m_1 ( S+ \eta R ) \Big ) + \frac{ca_3^l}{a_3^l + E^l} S \Big ) \nonumber \\&\qquad + \lambda _3 \Big ( (1-u) r F - \mu E \Big ) + \lambda _4 \Big (k_2 F (1-m_2 F) - \alpha (S+R)F \Big ), \end{aligned}$$

and $\xi _i(t) \ge 0$ are penalty multipliers such that

4.11 $$\begin{aligned} \xi _1(t) (u - u_a) =0, \qquad \xi _2(t)(u_b-u) = 0 \hbox { at } u^{*}. \end{aligned}$$

From the Pontryagin’s Maximum Principle, we can derive the adjoint equations by obtaining partial derivative of the model (3.1) with respect to *S*, *R*, *E* and *F*, respectively. Indeed, we get

4.12 $$\begin{aligned} \frac{d \lambda _1}{dt}&= - \frac{\partial {\mathcal {L}}}{\partial S}, \qquad \frac{d \lambda _2}{dt} = - \frac{\partial {\mathcal {L}}}{\partial R}, \nonumber \\ \frac{d \lambda _3}{dt}&= - \frac{\partial {\mathcal {L}}}{\partial E}, \qquad \frac{d \lambda _4}{dt} = - \frac{\partial {\mathcal {L}}}{\partial F}, \end{aligned}$$

with $$\lambda _i(t_{f}) = 0, i=1, \ldots , 4$$.

To obtain an expression of the control, we differentiate the Hamiltonian with respect to *u* as

4.13 $$\begin{aligned} \frac{\partial {\mathcal {H}}}{\partial u} = \omega _u u - r F \lambda _3, \end{aligned}$$

and project it onto the admissible set of controls. $\square$

### Implementation of the Optimal Control Problem

Third-generation AIs (anastrozole, letrozole and exemestane) reduce whole-body aromatisation by >90% [summarised in ref. Geisler and Lønning (2005)]. However, limited local estrogen production in tissue compartments cannot be totally ruled out. Also, it is possible that some cells could locally produce some estrogen under treatment (Sasano et al. 2009; Geisler 2003). Therefore, we assume that the maximum drug dose does not eliminate the total estrogen in the vicinity of tumor. This could be done simply by setting a threshold value on the control function. We use $u_a = 0$ and $u_b = 0.99$, where $u_a$ corresponds to the case of no treatment and $u_b$ refers to the strongest possible treatment.

The optimality system consisting of the state equation (4.2), the adjoint equation (4.7) and the optimality condition (4.8) form a nonlinear system of equations, so we obtain the numerical solution via forward-backward sweep (FBS) method (Lenhart and Workman 2007). As explained by Lenhart and Workman (2007), the FBS method requires initiation of a feasible control function to solve the state equation forward in time. Then, the adjoint equation is solved backward in time and the optimality condition is updated at each iteration until the stopping criterion is satisfied. Here, the idea is to find a feasible optimal control iteratively. The update strategy of the control could be done in different ways such as taking average of the current ($u_{cur}$) and previous control ($u_{pre}$) or their convex combination (Lenhart and Workman 2007). Here, we apply the approach "greedy" convex combination studied by Vatcheva et al. (2021, Sect. 3) to cover a large range of control combinations during optimization and avoid stagnation. "greedy" convex combination refers to expressing the control as $u_s = (1-s) u_{pre} + s u_{cur}$ where $s \in (0,1)$ is selected in such a way that the smallest value of ${\mathcal {J}}(u_s)$ is achieved in that iteration. The parameter *s* is not fixed as opposed to the averaging or convex combination, it may vary in each iteration instead. The stopping criterion in this paper is based on the relative error of the current and previous state, adjoint and control functions. The program is terminated when a relative error less than $10^{-5}$ is achieved.

Simulations in this study were performed using MATLAB^®^ R2022 (MATLAB 2022). We used ode15s solver to obtain the numerical solution of the differential equations and fmincon function in the model calibration step. All data and code are available (see data and code availability part for the details).

## Simulation Results

We focus in simulations of the extended model (3.1) that explore the effect of threshold values $a_2$ and $a_3$. These two values correspond to the estrogen concentrations below which cancer cells die or become resistant, respectively. Thus, simulation scenarios using different threshold values represent treatment in hypothetical tumors with differential sensitivities and rates of resistance to the local estrogen availability. For each case, we simulate three different treatment types: constant treatment, alternating treatment and optimal anti-hormonal treatment. We use the parameter values which are common in both the first and the extended model. For the others, we either fix their values or explore their impact in simulations. We list all parameter values in Table 2.

**Table 2:** Values of the parameters in the Model (4.1)–(4.2)

Parameter	Description	Units	Value
$k_1$	Growth rate of sensitive cells	day$^{-1}$	0.55 (calibrated)
$\mu$	Estrogen washout rate	day$^{-1}$	5.94, Deshpande et al. (1967)
$\eta$	Population competition intensity	–	1 (assumed)
$m_1$	Inverse carrying capacity of tumor	mm$^{-3}$	1/2000 (assumed)
*c*	Death rate	day$^{-1}$	1 (assumed)
*l*	Hill’s coefficient	–	10 (assumed)
$a_1$	Half maximum estrogen threshold	pg/g	43 (calibrated)
$a_2$	Estrogen threshold for sensitive cells to die	pg/g	Varies
$a_3$	Estrogen threshold for conversion to resistant	pg/g	Varies
$k_3$	Growth rate of resistant cells	day$^{-1}$	Varies
*p*	Effect of treatment	–	Varies
$k_2$	Fat growth rate	day$^{-1}$	0.05 (assumed)
$m_2$	Inverse carrying capacity of fat	mm$^{-3}$	0.002711$^{1}$
*r*	Estrogen production rate	pg/g mm$^{-3}$ day$^{-1}$	20 (assumed)
$\alpha$	Fat consumption rate	day$^{-1}$ mm$^{-3}$	1.7e$-$06 (calibrated)
$t_f$	Final time	Days	25
$u_a$	Minimum treatment	–	0
$u_b$	Maximum treatment	–	0.99
$\omega _S, \omega _R, \omega _u$	Positive weight coefficients	–	1$^{2}$

$^{1}$See appendix for computational details

$^{2}$unless otherwise stated

For constant treatment, the parameter *p* is chosen from the set $\{ 1, 0.025, 0.0125, 0.01, 0.001 \}$. For alternating treatment, we set $u_b=0.99$. Treatment is started on the date corresponding to the earliest time point $t:=t_{tr}$ at which $S + \eta R < \frac{1}{4 m_1}$ so that the tumor reaches a detectable size. The final simulation time is fixed as $t_f=25$ to obtain a unique optimal control [see (Fister et al. 1998, Sect. 4), for detailed discussion].

We choose the weight coefficients $\omega _S, \omega _R, \omega _u$ in the cost functional as one or hundred to model different penalization strategies. For instance, values $\omega _S, \omega _R > \omega _u$ refers to penalization of tumor cells more than treatment cost.

### Scenario I with $a_2=20$ pg/g, $a_3=1$ pg/g, $k_3 = \frac{k_1}{2}$

#### Scenario Ia: Adaptive resistance with $R_0=0$

We first explore a scenario without preexisting resistance, where initially all cells are assumed to be sensitive to treatment and no resistant cells exist. Instead, endocrine resistance may arise due to adaptation to low estrogen levels. Tumor cells may die if estrogen level is below $a_2=20$ pg/g and they may become resistant if estrogen concentration falls below $a_3=1$ pg/g. Proliferation rate of the sensitive cells is assumed to be equal to half of the growth rate of sensitive cells.

**Fig. 3:**
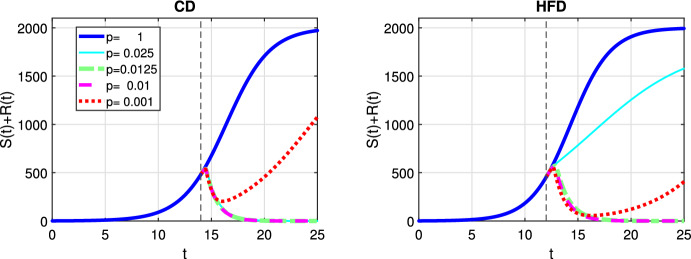
Scenario Ia: Sum of the sensitive *S* and resistant *R* tumor subpopulations over time *t* for the constant treatment with different values of *p* associated with CD (*left*) and HFD (*right*). We mark the time point at which treatment is started with a dashed vertical line (color figure online)

In Fig. 3, we show response to constant treatment by displaying the change in tumor size for different values of *p*. The solid line corresponds to the case where no treatment is applied, i.e., $p=1$ and tumor reaches to the carrying capacity as time passes. We mark in the figures the time point at which treatment is started with a dashed vertical line and we observe that treatment is started earlier for HFD than CD. We observe that anti-hormonal treatment results in eradication of tumor for the values $p=0.0125$ and 0.01 for both CD and HFD, whereas the case $p=0.025$ does not lead to tumor eradication for HFD. In case of a drug inhibiting estrogen production 99.9%, i.e., $p=0.001$, drug resistance is observed.

**Fig. 4:**
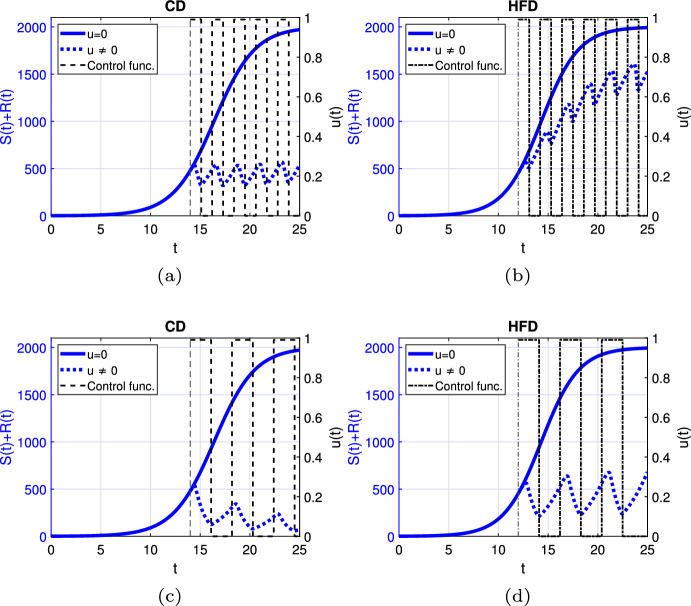
Scenario Ia: Left axis refers to the sum of the sensitive *S* and resistant *R* tumor subpopulations over time *t* for alternating treatment with **a**–**b** shorter phases (one day), **c**–**d** longer phases (two days) associated with CD (*left*) and HFD (*right*); right axis refers to treatment schedule. The dotted and solid curves refer to the tumor size with and without treatment, respectively. We mark the time point at which treatment is started with a dashed vertical line (color figure online)

Next, we proceed with alternating treatment. We simulate two different schedules with long and short treatment phases in Fig. 4. The solid curve refers to the tumor size without treatment. For CD, alternating treatment with shorter phases causes tumor volume to stay within a range. Instead for HFD, tumor size grows over time with respect to the baseline tumor volume. On the other hand, treatment with longer phases leads to tumor reduction for CD, while it stays within a range for HFD.

**Fig. 5:**
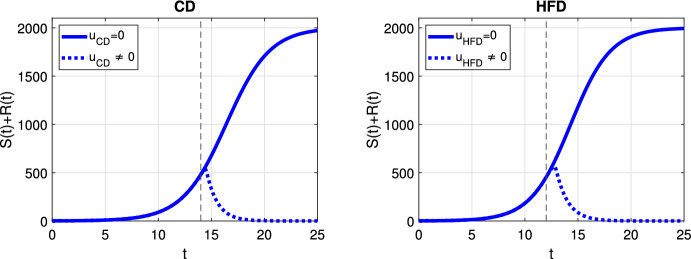
Scenario Ia: Sum of the sensitive *S* and resistant *R* tumor subpopulations over time *t* for the optimal treatment with different values of *p* associated with CD (*left*) and HFD (*right*). The solid curve refers to the tumor size without treatment. We mark the time point at which treatment is started with a dashed vertical line (color figure online)

**Fig. 6:**
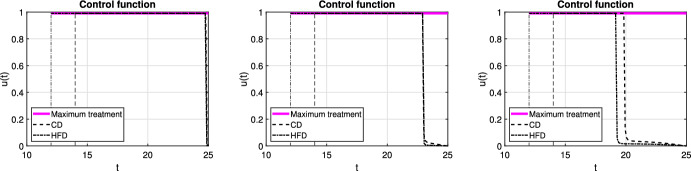
Scenario Ia: Optimal control function *u* over time *t* for three different combinations of weight coefficients $\omega _R, \omega _S, \omega _U$. Dashed and dash-dotted curves refer to the optimal treatment schedules for CD and HFD, respectively. Solid line denotes the maximum treatment. We mark the time point at which treatment is started with dashed and dash-dotted vertical lines for CD and HFD, respectively (color figure online)

**Fig. 7:**
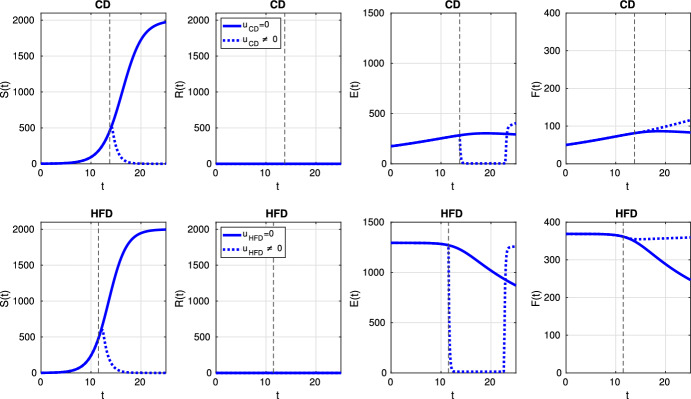
Scenario Ia: Dynamics of model variables *S*, *R*, *E* and *F* over time *t* associated with CD (*1st row*) and HFD (*2nd row*). The solid curve refers to the tumor size without treatment, dotted curve corresponds to results for optimal treatment (color figure online)

In case of optimal treatment scheduling, we observe in Fig. 5 that the tumors are eradicated for both CD and HFD (solid line for no treatment, dashed line for optimal treatment with $\omega _R= \omega _S = \omega _U = 1$). For comparison, optimal control functions *u*(*t*) are shown in Fig. 6 for different values of the weight constants. The case $\omega _R, \omega _S > \omega _U$ leads in both CD and HFD to maximum treatment for almost the entire studied period, whereas treatment could be stopped earlier if $\omega _R, \omega _S < \omega _U$. In other words, penalizing tumor cells more than treatment results in longer treatment. There is no big difference between control functions in terms of diet, except for a slightly larger duration of treatment for HFD. To observe the effect of treatment in more detail, we present in Fig. 7 the dynamics of all variables in the case $\omega _R= \omega _S = \omega _U = 1$. In this example, optimal treatment maintains the estrogen level between $a_3$ and $a_2$, so sensitive cells die, but no resistance occurs. This happens in spite of an increasing fat volume. Therefore, optimal treatment results in successful elimination of tumor without causing drug resistance.

**Fig. 8:**
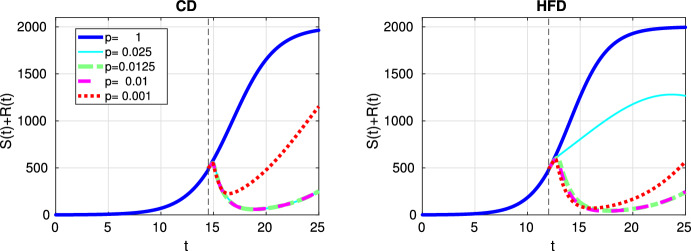
Scenario Ib: Sum of the sensitive *S* and resistant *R* tumor subpopulations over time *t* for the constant treatment with different values of *p* associated with CD (*left*) and HFD (*right*). We mark the time point at which treatment is started with a dashed vertical line (color figure online)

#### Scenario Ib: De novo resistance with $R_0=0.25$

Next we investigate the influence of a preexisting resistant sub-population on the success of constant, alternating and optimal anti-hormonal treatment schedules. In this case, endocrine resistance arise by clonal selection of cells that are endocrine independent for some reasons. In Fig. 8 we show response to constant treatment by displaying the change in tumor size for different values of *p*. We observe that constant treatment is unsuccessful to eliminate the tumor.

**Fig. 9:**
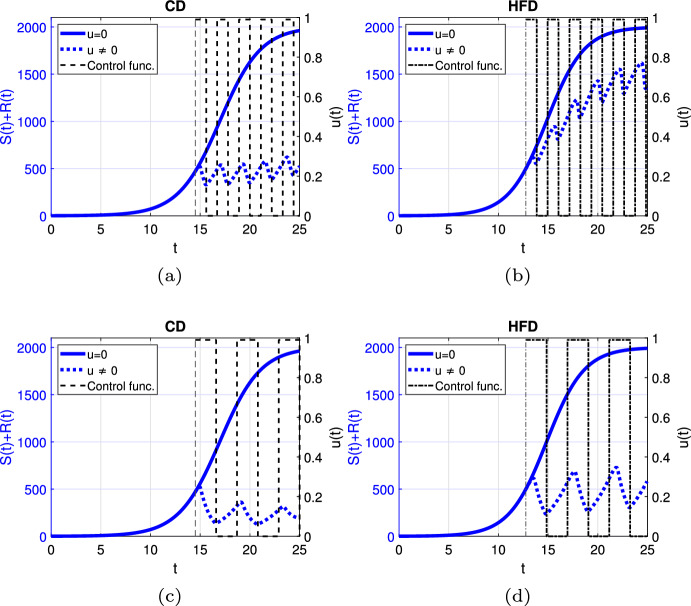
Scenario Ib: Left axis refers to the sum of the sensitive *S* and resistant *R* tumor subpopulations over time *t* for alternating treatment with **a**–**b** shorter phases (one day), **c**–**d** longer phases (two days) associated with CD (*left*) and HFD (*right*); right axis refers to treatment schedule. The dotted and solid curves refer to the tumor size with and without treatment, respectively. We mark the time point at which treatment is started with a dashed vertical line (color figure online)

We plot the change of tumor size over time for short and long alternating treatment phases in Fig. 9 where 75$\%$ of the cells are sensitive and 25$\%$ are resistant at the beginning of the simulation. For shorter drug holidays, tumor size increases compared to the initial tumor size. For longer on-off periods, the tumor volume remains within a bounded range for both CD and HFD but the oscillations between remission and growth are bigger in the HFD case. Moreover, the final tumor volume is larger for both cases in comparison with the case of no preexisting resistance showed in Fig. 4.

**Fig. 10:**
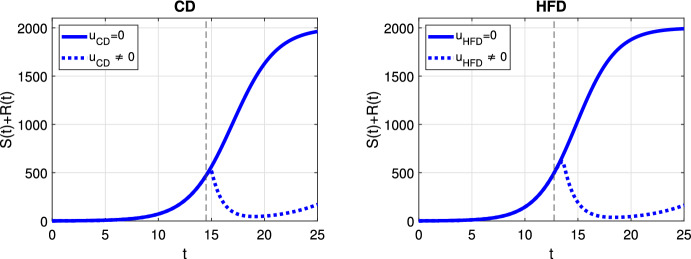
Scenario Ib: Sum of the sensitive *S* and resistant *R* tumor subpopulations over time *t* for the optimal treatment with different values of *p* associated with CD (*left*) and HFD (*right*). The solid curve refers to the tumor size without treatment. We mark the time point at which treatment is started with a dashed vertical line (color figure online)

**Fig. 11:**
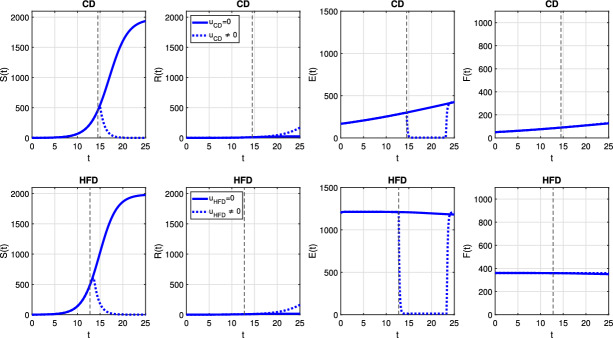
Scenario Ib: dynamics of model variables *S*, *R*, *E* and *F* over time *t* associated with CD (*1st row*) and HFD (*2nd row*). The solid curve refers to the tumor size without treatment, dotted curve corresponds to results for optimal treatment (color figure online)

We plot the results obtained with optimal treatment in Fig. 10. While treatment decreases the tumor volume in both CD and HFD cases, resistance cells proliferate and drug resistance occurs. We present the temporal evolution of the model variables in detail in Fig. 11. We can see that sensitive cells are killed but resistant cells increase in size as a result of drug-resistance. Optimal control profiles are similar to the case of adaptive resistance in Fig. 6, so we do not present it here.

### Scenario II: Adaptive resistance with $a_2=10$ pg/g, $a_3=1$ pg/g, $k_3 =\frac{k_1}{2}$

**Fig. 12:**
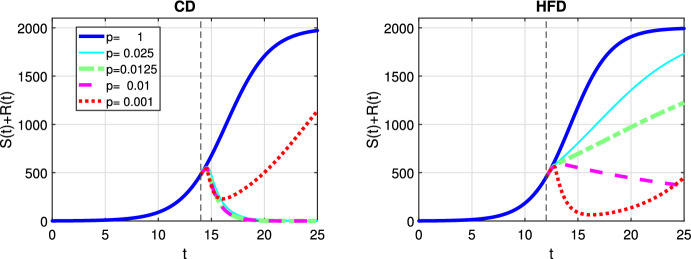
Scenario II: Sum of the sensitive *S* and resistant *R* tumor subpopulations over time *t* for the constant treatment with different values of *p* associated with CD (*left*) and HFD (*right*). We mark the time point at which treatment is started with a dashed vertical line (color figure online)

**Fig. 13:**
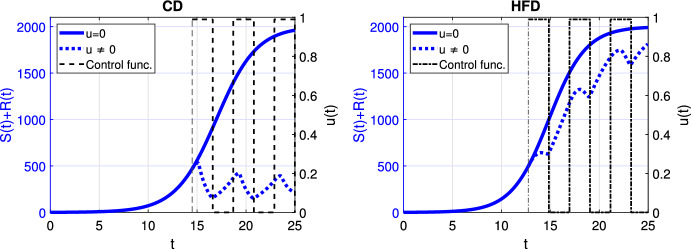
Scenario II: Left axis refers to the sum of the sensitive *S* and resistant *R* tumor subpopulations over time *t* for alternating treatment associated with CD (*left*) and HFD (*right*); right axis refers to treatment schedule. The dotte and solid curves refer to the tumor size with and without treatment, respectively. We mark the time point at which treatment is started with a dashed vertical line (color figure online)

Here we investigated a situation with no preexisting resistant cells but where the estrogen thresholds for which the cells die or are converted to resistant are closer than in the previous case. Figure 12 shows the case for constant treatment. All tested treatment cases, except $p=0.001$, result in tumor elimination for CD, but $p=0.01$ leads to tumor reduction until day 25 and higher values of *p* suppresses tumor growth for HFD. Alternating treatment instead, leads to oscillations in tumor size but with a decreasing trend for CD, whereas for HFD a sharp increase in tumor population is observed during drug holidays (see Fig. 13).

**Fig. 14:**
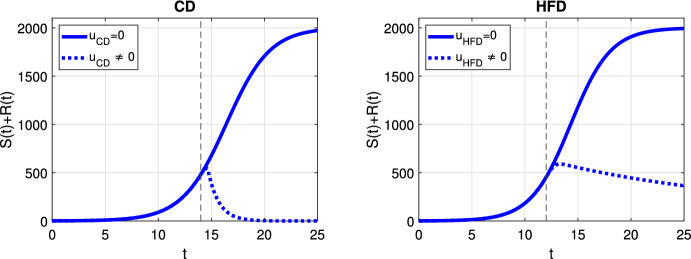
Scenario II: Sum of the sensitive *S* and resistant *R* tumor subpopulations over time *t* for the optimal treatment with different values of *p* associated with CD (*left*) and HFD (*right*). The solid curve refers to the tumor size without treatment. We mark the time point at which treatment is started with a dashed vertical line (color figure online)

The results for optimal treatment shown in Fig. 14 reveal that tumor is eradicated for CD, similar to Fig. 5. Interestingly, the tumor remains at the end of the treatment for HFD.

**Fig. 15:**
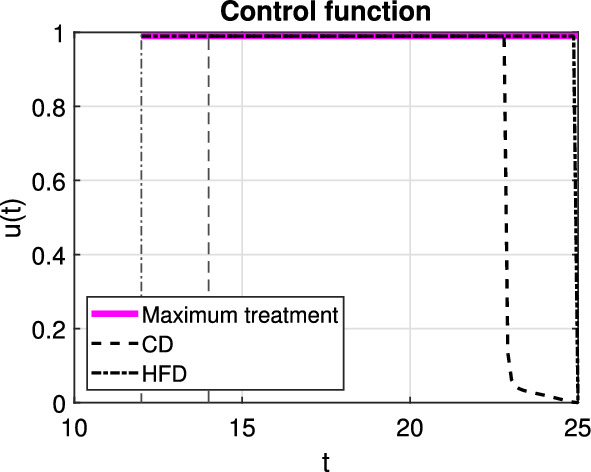
Scenario II: Optimal control function *u* over time *t* with $\omega _R=\omega _S=\omega _U=1$. Dashed and dash-dotted curves refer to the optimal treatment schedules for CD and HFD, respectively. Solid line denotes the maximum treatment. We mark the time point at which treatment is started with dashed and dash-dotted vertical lines for CD and HFD, respectively (color figure online)

We compare optimal treatment schedules in Fig. 15 for CD and HFD. We note that treatment must be applied for long time for HFD than CD, while it could be relaxed earlier for CD. Thus, optimal anti-hormonal treatment gives the most promising results among three different treatment choices in terms of reduction in tumor volume and time to lessen treatment could also be seen.

### Scenario III: Adaptive resistance with $a_2=a_3=10$ pg/g, $k_3 = \frac{k_1}{4}$

**Fig. 16:**
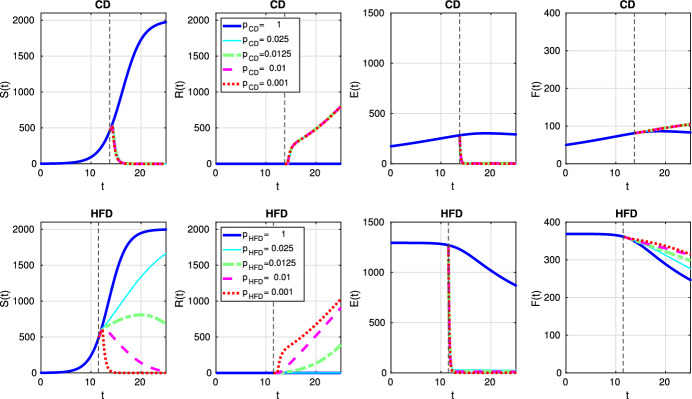
Scenario III: Dynamics of model variables *S*, *R*, *E* and *F* over time *t* associated with CD (*1st row*) and HFD (*2nd row*) (color figure online)

**Fig. 17:**
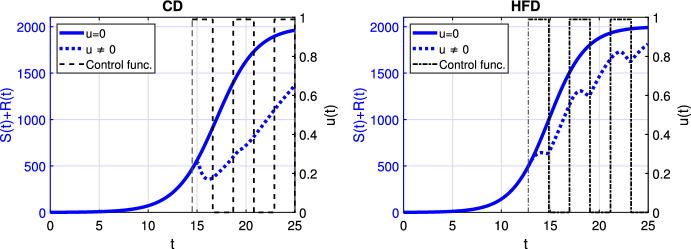
Scenario III: Left axis refers to the sum of the sensitive *S* and resistant *R* tumor subpopulations over time *t* for alternating treatment associated with CD (*left*) and HFD (*right*); right axis refers to treatment schedule. The solid line refers to the tumor size without treatment. We mark the time point at which treatment is started with a dashed vertical line (color figure online)

Finally, we investigate a scenario where death and conversion terms are equivalent, namely $a_2 = a_3 = 10$. We present temporal evolution of all model variables for constant treatment in Fig. 16. Estrogen level is successfully decreased, but it leads to drug resistance for CD for all choices of the parameter *p*. On the other hand, for HFD, the case $p=0.025$ is not strong enough to kill sensitive cells, so resistance cells do not proliferate. However, other treatment choices result in resistance and treatment fails. On the other hand, alternating treatment is not a successful strategy (see Fig. 17).

**Fig. 18:**
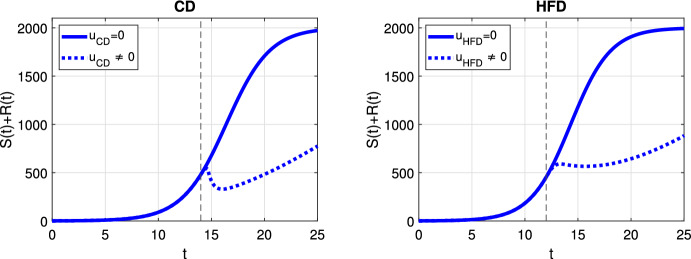
Scenario III: Sum of the sensitive *S* and resistant *R* tumor subpopulations over time *t* for the optimal treatment with different values of *p* associated with CD (*left*) and HFD (*right*). The solid curve refers to the tumor size without treatment. We mark the time point at which treatment is started with a dashed vertical line (color figure online)

**Fig. 19:**
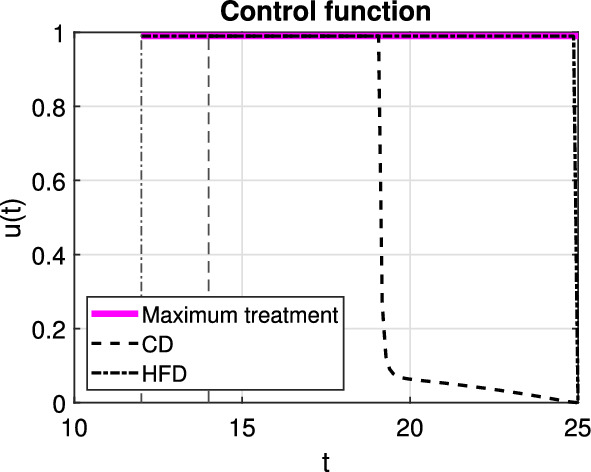
Scenario III: Optimal control function *u* over time *t* with $\omega _R=\omega _S=\omega _U=1$. Dashed and dash-dotted curves refer to the optimal treatment schedules for CD and HFD, respectively. Solid line denotes the maximum treatment. We mark the time point at which treatment is started with dashed and dash-dotted vertical lines for CD and HFD, respectively (color figure online)

Finally, optimal AI treatment results in drug resistance as seen in Fig. 18. Initial tumor size reduction is followed by cell proliferation. Even though treatment is stopped earlier for CD than HFD (see Fig. 19), it is not possible to eliminate resistance due to equal cell death and conversion terms in the model.

**Fig. 20:**
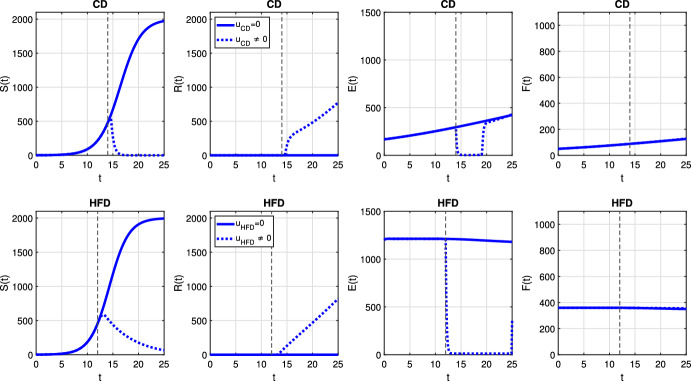
Scenario III: Dynamics of model variables *S*, *R*, *E* and *F* over time *t* associated with CD (*1st row*) and HFD (*2nd row*). The solid curve refers to the tumor size without treatment, dotted curve corresponds to results for optimal treatment (color figure online)

A detailed picture of model variables is presented in Fig. 20 and it reveals that treatment kills sensitive cells due to low estrogen level; but, then resistance occurs.

### Conclusions of the Simulation Results

We compared outcomes for different treatments in a series of hypothetical tumors with differential sensitivities and rates of resistance to the local estrogen availability. We observed that in tumors where the difference between estrogen thresholds for cancer cells to die and to adapt to low estrogen levels is large, then constant treatment with an appropriate dose or optimal treatment are the best for the case of only adaptive resistance. However, if the difference between the thresholds is smaller, then optimal treatments are better, specially in the HFD case. In case of preexisting resistance, if the difference between thresholds for cancer cells to die and to adapt to low estrogen levels is large, optimal treatment or constant treatment with appropriate dose gives the best outcome. When death of cancer cells and their adaptation to level of estrogen occur at the same threshold value, optimal treatment is best choice. Importantly, treatment outcome and optimal treatments schedules differ based on diet.

## Discussion

Given the rising obesity rates around the world, novel strategies are urgently needed to evaluate and optimise endocrine treatment of breast cancer in women with high BMI. In this study we focused on modeling the effect that fat-induced production of estrogen has on tumor growth. While our model is able to capture the trends in the experimental data for CD and HFD mice, we recognise that other factors associated with the adipose tissue and not considered in our current model, such as inflammatory cytokines, leptin or insulin, could be influencing tumour growth differently in the CD and HFD cases. These are subjects that deserve further investigation (Hillers-Ziemer et al. 2022).

By incorporating AI treatment and resistance in our model, we can simulate treatment outcomes in CD and HFD mice. However, as we do not have data on treatment, the choice of parameters related to sensitivity and resistant to treatment were made by explorative simulations. For instance, we assumed cost of resistance in the sense that the growth rate of resistance cells is smaller than the growth rate of sensitive cells. Otherwise, rapidly increasing resistant cells would always dominate the tumor. In addition to this, more than two tumor subpopulations with differential drug-response to AI could exists. When AI treatment data in these mice are available, it would be possible to obtain the number of subpopulations, their fractions and their growth rates trough a novel phenotypic deconvolution method (Köhn-Luque et al. 2023).

Besides constant and alternating treatments, we investigated optimal scheduling trough OCPs. In this framework, we underline that one of the theoretical challenges is to prove uniqueness of the optimal control on a specific time interval $[0, t_f]$, since the value of $t_f$ cannot be found explicitly, and it is bounded by some constants depending on the solutions of the state and adjoint equation. We observe that a larger time interval leads to convergence issues, which is an indication of the uniqueness of the solution on a smaller time interval. We have also experienced that the more complicated the ODE model used in the OCP constraint is, the smaller the time interval where a unique solution can be found. Furthermore, uniqueness could be proved using constant tumor growth rate, but we believe this is not a correct representation of ER-positive tumor subtype.

Being a breast cancer modeling study with optimal control analysis, Oke et al. constructed a model of four variables (including normal cells, tumor cells, natural killer cells and estrogen concentration) with implementation of anti-cancer drugs and a ketogenic diet (Oke et al. 2018). They modelled the ketogenic diet as a parameter affecting tumor growth, while anti-cancer drug was modeled as an intervention strategy leading to tumor death, and estrogen concentration to decrease, so that suppression of immune cell activation was relaxed. In addition, optimal values of the parameters corresponding to anti-cancer drugs and ketogenic diet were searched to minimize the total tumor size and estrogen concentration on a prespecified time interval within a quadratic optimal control setting. The authors noted that activities of cancer cells are reduced with the introduction of a ketogenic diet and they underlined the risk of ketoacidosis as a results of too much ketogenic diet. The authors found based on stability analysis of tumor-free equilibrium point that tumor cells could be eliminated with treatment and ketogenic diet, if the reproduction number of the system was reduced to a value less than one. This is in contrast with our simulations, where HFD does not result in better treatment outcomes. Interestingly, it has been shown that different fat diets, i.e. based on olive vs corn oil, influence breast tumor growth and progression differently (Costa et al. 2004; Solanas et al. 2009), adding complexity to the challenge of optimizing breast cancer treatment and diet.

Overall, the most striking observations from our simulations are that optimal aromatase inhibitor treatment schedules and the corresponding outcomes differ based on diet, which suggests that low fat diet and other measures to reduce the amount of fat could be introduced to improve treatment outcomes in obese patients. In our ongoing studies, we are modeling such patient-specific treatments making use of individual level data from the NeoLetExe trial (Bahrami et al. 2019), a neoadjuvant study exploring the lack of cross-resistance between the aromatase inhibitor letrozole and the aromatase inactivator exemestane. The effect of switching to a low-fat diet is not necessarily immediate, because it also depends on the lifestyle and how the body is prone to accumulate fat. Although our extended model might be able to capture lifestyle effects different than diet, this remains to be investigated.

### Supplementary Information

Below is the link to the electronic supplementary material.

Supplementary file 1 (pdf 194 KB)

## Data Availability

Simulations in this study were performed using MATLAB^®^ R2022 (MATLAB 2022). All data and code used in this article are publicly available in the online repository of the Oslo Center for Biostatistics and Epidemiology (OCBE).
