# Supplementary material for: Modeling of Mouse Experiments Suggests that Optimal Anti-Hormonal Treatment for Breast Cancer is Diet-Dependent
Source: Bull Math Biol. 2024 Mar 18;86(4):42. doi: 10.1007/s11538-023-01253-1 (PMC11310292; doi:10.1007/s11538-023-01253-1)
Supplement: Supplementary file 1 — (pdf 194 KB) [file 11538_2023_1253_MOESM1_ESM.pdf]

7 Supplementary Material

Computation of fat volume at $t = 15$ and carrying capacity of adipocytes: Growth of adipose tissue is based on two processes affected by genetic and diet differences, namely cell number increase (hyperplasia) and cell size increase (hypertrophy) (Jo et al. 2009). Therefore, computation of the amount of fat at days $t = 0$ and $t = 15$ in the system are based on these two mechanisms. We start by calculating the average number of adipocytes per image and per mm^2 for CD and HFD, separately, based on the study (Hillers et al. 2018, Fig. S2F).

On the other hand, Bozec and Hannemann report that adipocyte size increases in mice fed with HFD, so we set the diameters of adipocytes for CD and HFD as 0.1 mm and 0.2 mm, respectively, as reported in the study (Bozec and Hannemann 2016, Fig.6C)which leads to two different values for the amount of fat at time $t = 15$ for CD and HFD. In addition, mice are fed with control diet after tumor injection in the experiment, so we assume that carrying capacity of adipocytes associated with mice fed with CD and HFD are determined by the amount of fat for HFD at $t = 15$.

Procedure to estimate the amount of fat in the cube with volume V could be summarized as follows: Let n be the number of adipocytes per mm^2 and d be the average diameter (mm) of an adipocyte. Then, $\sqrt[3]{V}$ is the size of the cube in mm with volume V and $\frac{\sqrt[3]{V}}{d}$ is the number of layers with height d in the cube, where d is the diameter of an adipocyte. In addition, we compute $\sqrt[3]{V} \times n$ adipocytes per mm^2. Finally, the approximate number of adipocytes in the cube with volume V could be aproximated via the product $\frac{\sqrt[3]{V}}{d} \times \sqrt[3]{V} \times n = \frac{n\sqrt[3]{V^2}}{d}$.

Profile likelihood calculations We present profile likelihood calculations for model (2.1) after performing calibration (Kreutz et al. 2012) in Fig. 21. We used "arPLEInit" and "ple" functions of d2d software. The figure indicates practical identifiability of the model corresponding to the 95% confidence level for the parameters a_1 and k_1. For parameter α, this threshold is above 83%.

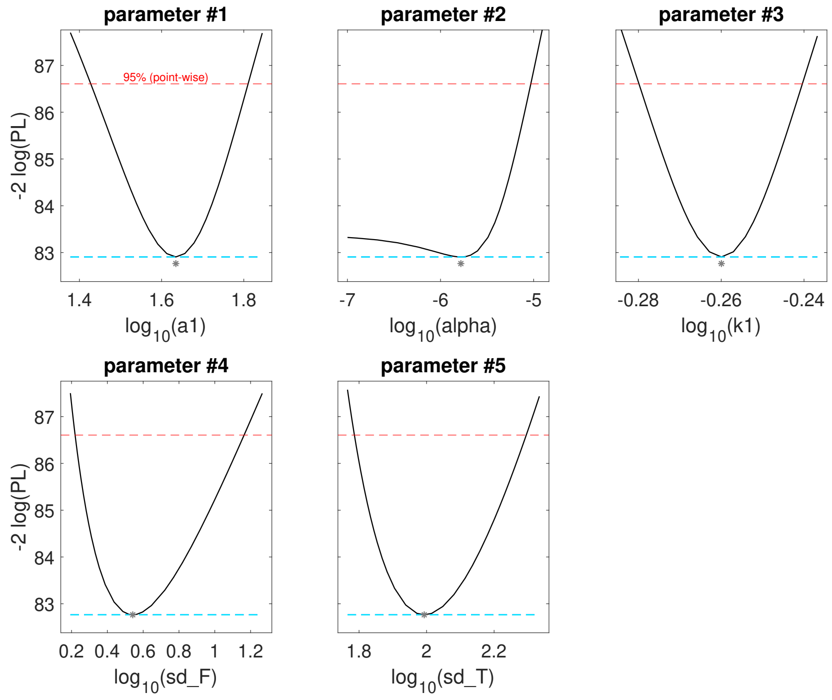

Fig. 21: Profile likelihood computations for the calibrated parameters a_1, α, k_1.

Sensitivity analysis

We present the results of the global sensitivity analysis for Eq. 2.1 to capture the relative changes of the variables with respect to the parameters at days 5, 15 and 25. Here, the method of partial rank correlation coefficient (PRCC) presented by Marino et al. is used (Marino et al. 2008).

Global sensitivity analysis requires a uniformly distributed sample space which is constructed for each parameter with 1000 sample values. Here, intervals for each parameter are constructed with the end points as $\pm$ 5 % of the baseline parameter. LHS-PRCC MATLAB code given in the web site (Lab 2020) have been used and modified for the current model. The idea behind PRCC is to assign a value between -1 and +1 to each relation. The magnitude of this value determines the strength and the sign indicates the trend of the relation between the parameter and the variable. We briefly compare the most sensitive parameters where sensitivities for CD and HFD are grouped on the left and right panel in Fig. 22. Firstly, we observe that parameters k_1 and r have positive sensitivities for tumor volume, whereas negative sensitivity with respect to μ decreases over time in magnitude. Sensitivity of estrogen concentration is positive for r and negative for μ, as expected. In addition, fat volume is negatively sensitive to k_1 and positive sensitivity with respect to m_1 increases over time and it is effect is smaller for HFD than CD case.

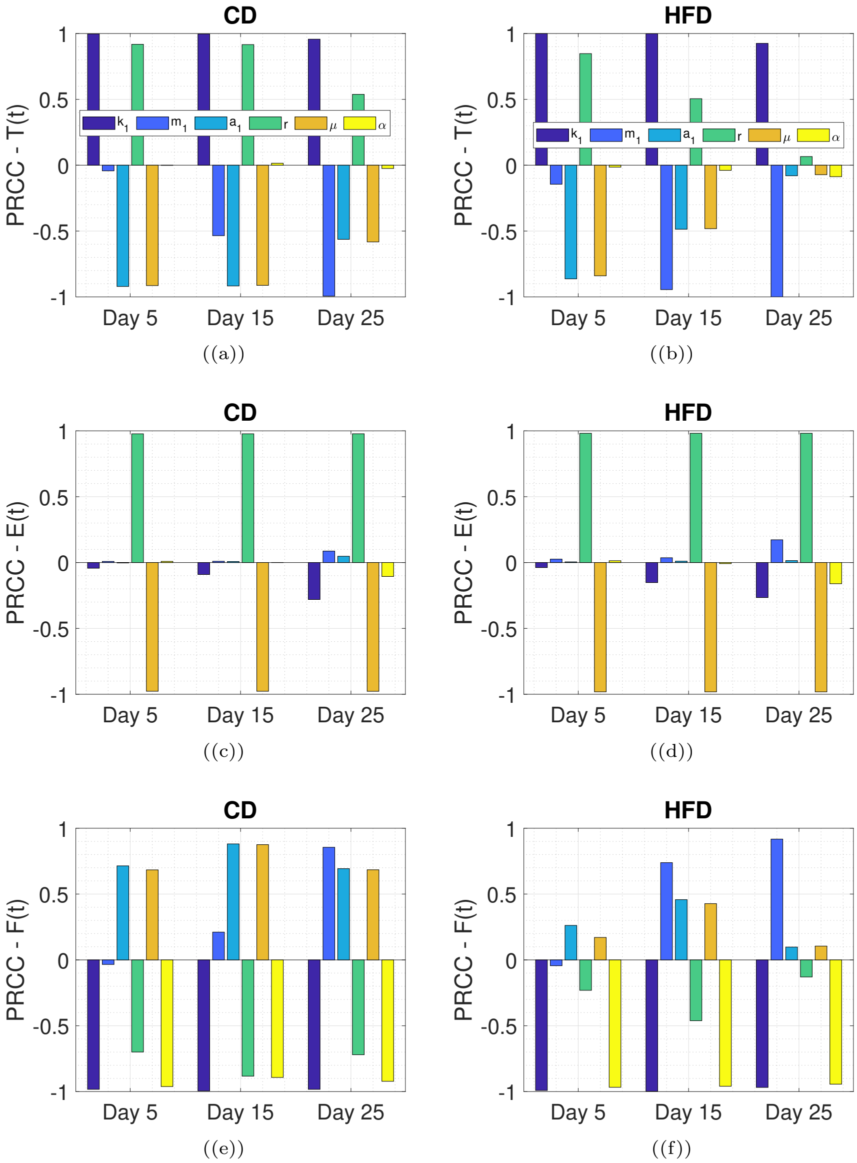

Fig. 22: Sensitivity analysis for CD (*left column*) and HFD (*right column*) for Eq. 2.1 at days 5, 15 and 25 where ((a)) - ((b)) T(t), ((c)) - ((d)) E(t), ((e)) - ((f)) F(t).
